# Adiponectin Modulates Smooth Muscle Cell Morpho-Functional Properties in Murine Gastric Fundus via Sphingosine Kinase 2 Activation

**DOI:** 10.3390/life13091812

**Published:** 2023-08-26

**Authors:** Rachele Garella, Caterina Bernacchioni, Flaminia Chellini, Alessia Tani, Francesco Palmieri, Martina Parigi, Daniele Guasti, Emanuele Cassioli, Giovanni Castellini, Valdo Ricca, Daniele Bani, Chiara Sassoli, Chiara Donati, Roberta Squecco

**Affiliations:** 1 Department of Experimental and Clinical Medicine, Section of Physiological Sciences, University of Florence, 50134 Florence, Italy; francesco.palmieri@unifi.it (F.P.); roberta.squecco@unifi.it (R.S.); 2 Department of Experimental and Clinical Biomedical Sciences “Mario Serio”, University of Florence, 50134 Florence, Italy; chiara.donati@unifi.it; 3 Department of Experimental and Clinical Medicine, Section of Anatomy and Histology, Imaging Platform, University of Florence, 50134 Florence, Italy; flaminia.chellini@unifi.it (F.C.); alessia.tani@unifi.it (A.T.); martina.parigi@unifi.it (M.P.); daniele.guasti@unifi.it (D.G.); daniele.bani@unifi.it (D.B.); chiara.sassoli@unifi.it (C.S.); 4 Psychiatry Unit, Department of Health Sciences, University of Florence, 50134 Florence, Italy; emanuele.cassioli@unifi.it (E.C.); giovanni.castellini@unifi.it (G.C.); valdo.ricca@unifi.it (V.R.)

**Keywords:** adiponectin, sphingosine kinase, smooth muscle, gastric fundus, morphology, membrane properties, signaling pathway

## Abstract

Adipokines are peptide hormones produced by the adipose tissue involved in several biological functions. Among adipokines, adiponectin (ADPN) has antidiabetic and anti-inflammatory properties. It can also modulate food intake at central and peripheral levels, acting on hypothalamus and facilitating gastric relaxation. ADPN exerts its action interacting with two distinct membrane receptors and triggering some well-defined signaling cascades. The ceramidase activity of ADPN receptor has been reported in many tissues: it converts ceramide into sphingosine. In turn, sphingosine kinase (SK) phosphorylates it into sphingosine-1 phosphate (S1P), a crucial mediator of many cellular processes including contractility. Using a multidisciplinary approach that combined biochemical, electrophysiological and morphological investigations, we explored for the first time the possible role of S1P metabolism in mediating ADPN effects on the murine gastric fundus muscle layer. By using a specific pharmacological inhibitor of SK2, we showed that ADPN affects smooth muscle cell membrane properties and contractile machinery via SK2 activation in gastric fundus, adding a piece of knowledge to the action mechanisms of this hormone. These findings help to identify ADPN and its receptors as new therapeutic targets or as possible prognostic markers for diseases with altered energy balance and for pathologies with fat mass content alterations.

## 1. Introduction

Adipokines are adipocyte-derived secretory protein factors implicated in a broad range of biological functions. Acting through autocrine, paracrine and endocrine mechanisms, they take part in several physiological and pathological phenomena, such as energy metabolism, cardiovascular and skin disease, polycystic ovary syndrome, ovarian cancer, endometriosis, etc. [1,2,3,4,5]. Among adipokines, adiponectin (ADPN) has been demonstrated to also have pleiotropic effects, promoting insulin sensitivity, regulating energy metabolism and cell survival and having anti-inflammatory [6] and antidiabetic properties [7]. In addition, it has been shown to also play a role in the regulation of food intake at the central level acting on pro-opiomelanocortin (POMC) and neuropeptide Y (NPY)-secreting neurons in the hypothalamic arcuate nucleus [8,9,10]. In addition to many beneficial systemic repercussions, ADPN has been demonstrated to exert a neuromodulatory role in strips from the mouse gastric fundus by means of the classical inhibitory mediator nitric oxide (NO) [11], facilitating smooth muscle relaxation and increasing the gastric wall distention. Notably, this effect could be seen as a reinforcing peripheral satiety signal in the complex regulation of food intake. A number of early papers focused on the effects of ADPN on gastric murine smooth muscle cells (SMCs) membranes aiming to elucidate its mechanism of action. In particular, it was recently demonstrated that ADPN strongly influences SMC properties by inducing several modifications of membrane features that hamper cell excitability such as hyperpolarization [12], increase of K^+^ current amplitude and decrease of connexin 43 (Cx43) expression [13]. Moreover, ADPN reduces calcium current (I_Ca_) amplitude, supporting the inhibitory effect on Ca^2+^ influx through voltage-dependent Ca^2+^ channels, further supporting its influence in hindering the SMCs’ electromechanical coupling [14].

In keeping with the molecular mechanism of action, it is known that ADPN exerts its effects mainly acting on two well-characterized and ubiquitously expressed seven transmembrane domains receptors, Adiponectin receptor 1 (AdipoR1) and Adiponectin receptor 2 (AdipoR2). They represent the major membrane receptors found in vivo mediating the actions of such a pleiotropic hormone [15] and their presence has been demonstrated in the brain [16] and in many mammalian peripheral tissues [17,18,19]. In particular, the expression of AdipoR1 and AdipoR2 was revealed by polymerase chain reaction (PCR) analysis also in the murine stomach [11]. A later study showed, by immunofluorescence labeling, AdipoR1 expression on glial cells associated with neurons in the myenteric plexus in strips of murine gastric tissue [12]. In the same study, the effects of ADPN as a prorelaxant hormone were shown to involve AMP-activated protein kinase (AMPK)/nitric oxide synthase (NOS)/NO on the murine gastric fundus [12]. Very recently, the effects of this adipokine on membrane excitability and K^+^ currents have been demonstrated to recruit the NO/guanylate cyclase (GC) pathway [13], adding further elucidation to this signaling pathway.

Of note, it has been reported that AdipoR1 and AdipoR2 are associated in various tissues with ceramidase activity that enhances the elimination of fatty acyl chain from ceramides and determines the generation of sphingosine. In turn, thanks to sphingosine kinase (SK) activity, sphingosine is phosphorylated to sphingosine-1-phosphate (S1P) by the specific enzyme. The result is a decrease in levels of ceramide and an increase in S1P following the binding to ADPN, highlighting the sphingolipid cascade as a crucial player of ADPN signaling path [6]. Interestingly, S1P is a well-known pleiotropic molecule that modulates cell proliferation, differentiation, survival, migration [20] and muscle cell biology [21,22,23,24]. Its production occurs in response to extracellular stimuli, namely growth factors and cytokines. Intracellular levels of S1P are finely regulated by SK, S1P-specific phosphatases and S1P lyase. In this way, the cell maintains S1P concentration at optimal levels and generates space–time variations in response to appropriate stimuli [25,26]. SK exists in two isoforms, SK1 and SK2 [27]. Although the two isoforms have a high homology rate and the same reaction product, they differ in subcellular localization and catalytic properties. Their activation depends on their phosphorylation, which is essential for the enzyme translocation at the plasma membrane level, where its substrate sphingosine is located [28,29].

Accordingly, based on previous papers reporting that ADPN pleiotropic effects involve ceramidase activity of AdipoR [6], and aiming to add a piece of knowledge to the possible mechanisms of action of this adipokine in modulating gastric fundus activity, the present study wished to investigate for the first time the possible role of the S1P pathway in mediating ADPN effects on murine stomach ex vivo preparations. To this end, we performed biochemical, electrophysiological and morphological investigations and, thanks to this multidisciplinary approach, we showed that ADPN effects on SMC membrane properties and contractile machinery involve the SK pathway also in the murine gastric fundus. These findings help to extend knowledge to identify ADPN and its receptors as potential novel therapeutic targets or prognostic markers for a number of diseases characterized by altered energy balance and for pathologies with fat mass content alterations.

## 2. Materials and Methods

### 2.1. Animals and Gastric Fundus Muscle Strip Preparation and Treatments

C57BL/6 (Charles River, Lecco, Italy) 8/12 months female mice were employed for the experiments, fed with standard laboratory food and water weeks and subjected to a photoperiod of 12 h of light and 12 h of dark at a controlled temperature (21 °C). For stomach explants, mice were sacrificed by quick cervical dislocation to minimize suffering. After the stomach removal from the abdomen, we excised the gastric fundus, which was incised along the lesser curvature and opened. Then, we longitudinally cut 2 or 3 full-thickness (2 × 10 mm) strips from this part of gastric tissue From each stomach, some strips were used as CTRL and others were subjected to the following treatments (at room temperature bubbled with 95% O_2_—5% CO_2_): ADPN (20 nM) for at least 20 min; SK2 inhibitor, SLC5111312, (SLC, 1 µM) for 20 min. To test the ADPN effect in the presence of the SK2 inhibitor, we first treated the sample with SLC5111312 1 µM for 20 min and then we added ADPN 20 nM for at least 20 more min. The drug concentrations and times of exposure used were chosen based on the effectiveness proven in previous studies on gastric preparations [13]. The concentrations are given as the final concentration in the bath. The experimental solutions were made before conducting the experiment. ADPN stock solution was kept at −20 °C. The chemicals used in our experiments were purchased from Sigma Chemical (Milan, Italy).

### 2.2. Western Blot Analysis

Tissues were dispersed in a buffer containing 50 mM Tris, pH 7.5, 120 mM NaCl, 1 mM EDTA, 6 mM EGTA, 15 mM Na_4_P_2_O_7_, 20 mM NaF, 1% Nonidet and protease inhibitor cocktail and disrupted in a Dounce homogenizer (100 strokes). Lysates, obtained following centrifugation (10,000× *g* for 15 min at 4 °C) at were resuspended in Laemmli sample buffer (Bio-Rad Laboratories, Hercules, CA, USA) and loaded on 4–20% pre-cast-SDS-PAGE gels (Bio-Rad Laboratories, Hercules, CA, USA) before being blotted onto PVDF membranes. Primary antibodies were added to the PVDF membranes overnight at 4 °C, while the horseradish peroxidase-conjugated secondary antibodies were added for 1 h at room temperature. Enhanced chemiluminescence reagent (GE Healthcare Europe (Milan, Italy) was employed to detect immunoreactive bands by Amersham Imager 600 (GE Healthcare, Chicago, IL, USA). Densitometric analysis was made by ImageJ software version 2.0.0-rc-64. The band intensities were normalized on the housekeeping gene GAPDH expression from the same PVDF membrane. Anti-phospho-SK2 (Thr578) (P-SK2) and anti-phospho-SK1 (Ser225) (P-SK1) antibodies were bought from ECM Biosciences LLC (Versailles, KY, USA). Monoclonal anti-AdipoR1, anti-AdipoR2, anti-GAPDH and secondary antibodies conjugated to horseradish peroxidase, were acquired from Santa Cruz Biotechnology (Santa Cruz, CA, USA).

### 2.3. Electrophysiological Experiments

For electrophysiological recordings, at least 5 mice were used. Each excised stomach was cleaned with Krebs–Henseleit solution (KH) [13] consisting of 118 mM NaCl, 4.7 mM KCl, 1.2 mM MgSO_4_, 1.2 mM KH_2_PO_4_, 25 mM NaHCO_3_, 10 mM glucose, and 2.5 mM CaCl_2_ (pH 7.4 with NaOH). First, we fixed the part of the mucosa upwards to accurately remove the mucosa and submucosa under a dissecting microscope. The remaining tissue was fixed with the serous part upwards and the connective tissue was eliminated in order to expose the smooth muscle layer. The sample obtained was finally fixed with the serous part facing upwards, in the recording chamber (Sylgard-coated p35 Petri dish) and fundus strips were obtained from each gastric sample cutting the tissue longitudinally. During the experiments, the pinned tissue was continuously perfused with KH solution at a rate of 1.8 mL/min. A conventional high-resistance glass electrode was introduced into a smooth muscle cell of the longitudinal muscle layer for the intracellular recordings [30]. A vertical puller was used for producing the microelectrodes from borosilicate glass tubes. Microelectrodes were usually filled with the following internal solution (mM): KCl 130, NaH_2_PO_4_ 10, CaCl_2_ 0.2, ethylene-bis (oxyethylenenitrilo) tetraacetic acid (EGTA) 1, MgATP 5 and 4-(2-hydroxyethyl)-1-piperazineethanesulfonic acid (HEPES)/KOH 10 (pH 7.2 with tetraethylammonium hydroxide). Once filled, the resistance of the pipette was 60–70 MΩ. The pH was set to 7.4 with NaOH and to 7.2 with tetraethylammonium hydroxide for the external and pipette solution, respectively. To measure the RMP in the current-clamp experiments, we used the KH solution as a control bath solution. To record only the Ca^2+^ currents the microelectrodes were filled with the following solution (mM): 150 CsBr, 5 MgCl_2_, 10 ethylene-bis (oxyethylenenitrilo) tetraacetic acid (EGTA), and 10 (4-(2-hydroxyethyl)-1-piperazineethanesulfonic acid) (HEPES), and in the extracellular bath an external solution was used with a high concentration of Ca^2+^ and TEA but without Na^+^ and K^+^, containing (mM) 10 CaCl_2_, 145 TEABr and 10 HEPES. Nifedipine was used to test for the presence of L-type calcium current. Heptanol (1 mM), blocker of the gap junctions, was added to the bath when we wanted to avoid electrical coupling between the SMCs and to allow only the recording of the phenomenon evoked by the cell under examination [14,31]. For a correct comparison of the currents arising from cells having different volume/area, the current size was usually normalized for the linear capacitance, Cm. This parameter, in fact, is related to the cell surface area, being the specific membrane capacitance constant at 1 μF/cm^2^. The current–voltage relation (I–V plot) was obtained by reporting the mean value of the current amplitudes measured at the end of the pulse and normalized for cell capacitance as a function of each voltage step applied.

#### Stimulation Protocols

In the current–clamp mode of our amplifier, we recorded the RMP of the SMCs using a pulse waveform of I = 0 pA, before and after drug addition. We evaluated the SMC membrane passive properties (Rm, Gm and Cm) in voltage clamp condition, by applying two 75-ms voltage steps, starting from a holding potential (HP) of −70 mV ranging from −80 to −60 mV. The activation of voltage-dependent calcium channels was evoked in the SMCs held at HP = −80 mV, by applying 1 s long potential steps from −70 to 50 mV, in 10 mV-increments, and an interval of 20 s was given between one episode and another to allow recovery. Capacitive, linear leakage and voltage-independent currents were canceled directly online using the P/4 procedure.

### 2.4. Morphological Analyses

#### 2.4.1. Hematoxylin and Eosin (H&E) Staining

Gastric fundus muscle samples were embedded in paraffin (*n* = 3 each) after being fixed with 10% formalin in phosphate-buffered saline (PBS), dehydrated with a graded alcohol series and cleared in xylene. From each sample (*n* = 30 for each experimental condition), at least 10 sections (thickness 5 µm) were sliced and colored with H&E. Morphological analysis of the tissue was achieved by a light microscope (Leica DM4000 B) equipped with a DFC310 FX 1.4-megapixel digital color camera and software application suite LAS V3.8 (Leica Microsystems, Mannheim, Germany).

#### 2.4.2. Confocal Immunofluorescence Analyses

Confocal indirect immunofluorescence analyses were performed on three gastric fundus muscle strips for each experimental condition essentially as previously reported [13]. The following primary antibodies (overnight at 4 °C) were employed: mouse monoclonal anti-AdipoR1 (1:100; Santa Cruz Biotechnology ), mouse monoclonal anti-AdipoR2 (1:100; Santa Cruz Biotechnology) and mouse monoclonal anti-α-sma (1:100; Abcam, Cambridge, UK, Cat #ab7817, Lot# GR3246513, RRID AB_262054), and rabbit anti-Phospho-Myosin Light Chain (p-MLC) 2 (Ser-19) (1:100; Cell Signaling Technology, Danvers, MA, USA, Cat #3671, Lot #7, RRID AB_330248). The immunoreactions were revealed by using an anti-mouse Alexa Fluor 488-conjugated IgG (1:200; 1 h at room temperature, Molecular Probes, Eugene, OR, USA, Cat # A11001, Lot# 1752514, RRID:AB_2534069) or anti rabbit Alexa Fluor 488-conjugated IgG (1:200, 1 h at room temperature, Molecular Probes, Cat #A11034, Lot# 1,094,393 RRID:AB_2576217). We attained negative controls by replacing the primary antibody with non-immune serum. By omitting the primary antibody we evaluated the cross-reactivity of the secondary antibody. We used the fluorescent red dye Propidium iodide (PI, Molecular Probes, Cat # P1304MP) to put in evidence the nuclei. Accordingly, tissue specimens were treated with PI 1:100, at room temperature for 2 min. Samples were observed with a confocal microscope Leica TCS SP5 (Leica Microsystems), by means of an objective Leica Plan Apo 40xNA. Optical section series (1024 × 1024 pixels each, pixel size 204.3 nm, 209 × 209 µm optical square field, and 0.4 µm in thickness) were acquired every 0.6 µm and projected onto a single ‘extended focus’ image. Differential Interference contrast (DIC) images were simultaneously acquired with fluorescence images. Digitized images were subjected to densitometric analysis of p-MLC2 and α-sma fluorescent signal intensity by means of ImageJ software (Version 1.49 v, RRID:SCR_003070; NIH, Bethesda, MD, USA). For each confocal stack we analyzed five regions of interest (ROI; 25 × 25 µm), five for each experimental condition. The analyses were achieved in triplicate (*n* for each experimental point = ROI = 75).

#### 2.4.3. Transmission Electron Microscopy (TEM)

TEM analysis was performed essentially as reported previously [13]. Three gastric fundus muscle strips were processed and analyzed for each experimental condition: CTRL, ADPN, SLC, SLC + ADPN. The Ultrathin Epon 812-sections (60 nm thick, Sigma-Aldrich, St. Louis, MO, USA, Cat # 45345), contrasted with alkaline bismuth subnitrate and UranyLess EM stain (Electron Microscopy Sciences, Foster City, CA, USA, Cat # 22409), were analyzed using a Jeol 1010 electron microscope (Jeol, Tokyo, Japan) at 80 kV equipped with a digital camera. Jeol Veleda high-magnification images (×100,000) of longitudinally sectioned smooth muscle cells, 5 from each experimental condition, were used for morphometric analysis of the relative area of actin microfilaments using Fiji-ImageJ 1.53t image analysis software. In each image, 4 regions of interest, 40,820 nm^2^ each, were chosen at random and thresholded to only include microfilaments. 

### 2.5. Data Analysis and Statistical Tests

The mathematical and statistical analysis of the data obtained from the electrophysiological experiments was carried out with Microsoft Excel (Microsoft, Washington, DC, USA). To compare the means of two data sets, we used the Student’s *t* test and the test ANOVA with Bonferroni’s correction for multiple comparisons. The number of investigated SMCs is indicated by *n*. Microsoft Excel was used to generate graphs. The statistical analysis of the confocal immunofluorescence images was performed by one-way ANOVA with Bonferroni’s post hoc test for multiple comparisons. One-way ANOVA and Newman–Keuls multiple comparison post tests were used for TEM images’ analysis using Prism 5 software (GraphPad Software, San Diego, CA, USA). Values are indicated as mean ± SD or ±SEM. *p* ≤ 0.05 is meant to be statistically significant.

## 3. Results

### 3.1. ADPN Activates Sphingosine Kinase 2 (SK2) in the Murine Gastric Fundus Smooth Muscle Samples

**Figure 1:**
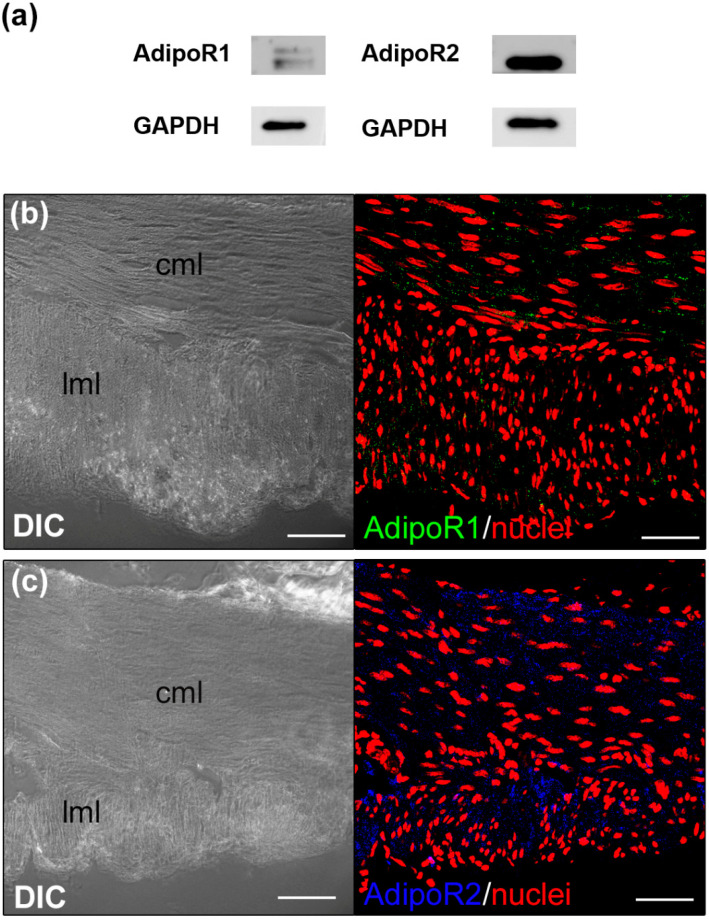
Adiponectin receptor 1 (AdipoR1) and 2 (AdipoR2) expression and localization. (**a**) Western Blot analysis performed in tissue lysates. (**b**,**c**) Representative differential interference contrast (DIC, grey) and confocal indirect immunofluorescence images of cross-section of paraffin-embedded samples stained with antibodies against AdipoR1 ((**b**), green) or AdipoR2 ((**c**), blue, pseudocolor). Nuclei are counterstained in red with Propidium iodide. Scale bar 50 μm. In (**b**,**c**): cml, circular muscle layer; lml, longitudinal muscle layer.

To clarify possible mechanisms of action of ADPN in gastric fundus, we first investigated the expression of AdipoR1 and AdipoR2 in the murine gastric fundus smooth muscle tissue. Western blot analysis revealed that both receptors were expressed in our samples (Figure 1a). In line with the biochemical results, confocal immunofluorescence analysis revealed the expression of the receptors both in the circular and in the longitudinal muscular layer. In particular, AdipoR2 seems to be highly expressed (Figure 1b,c).

**Figure 2:**
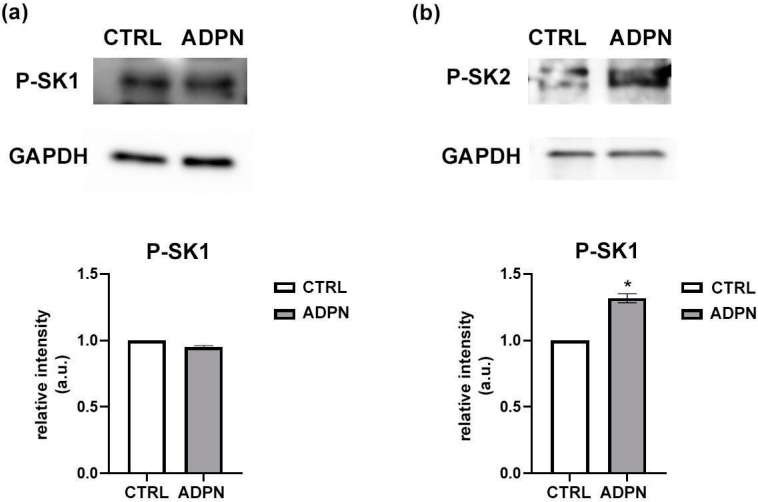
ADPN increases SK2 phosphorylation in murine gastric fundus. A fragment of stomach was treated with ADPN (20 nM) for 20 min before being lysed as described in the Methods Section (Section 2). Western blot analysis was performed using (**a**) specific anti-phospho-SK1 (Ser225) (P-SK1) and (**b**) anti-phospho-SK2 (Thr578) (P-SK2) antibodies. A blot representative of two independent experiments with analogous results is shown. The bar chart represents densitometric analysis of two independent experiments. Data are the mean ± SEM and are reported as protein expression normalized to GAPDH, fold change over control (set as 1). a.u.: arbitrary units. The increase in P-SK2 content induced by ADPN was found to be statistically significant by *t* test (* *p* < 0.05, treated vs. CTRL).

Subsequently, in order to test whether S1P signaling mediates ADPN action, we evaluated whether the adipokine was able to activate SK isoforms. Therefore, we quantified by Western Blot analysis the extent of the phosphorylated status of SK1 and SK2 (P-SK1, P-SK-2), hint of their activation, following ADPN stimulation. We found that ADPN treatment induced an increase by 30% in SK2 phosphorylation, without altering SK1 activity (Figure 2). This indicates that, although both SK isoforms are revealed in our gastric fundus samples, ADPN just promotes SK2 activation.

### 3.2. ADPN Effects on Membrane Properties Involve SK2 Signaling Pathway in the Murine Gastric Fundus SMCs

**Figure 3:**
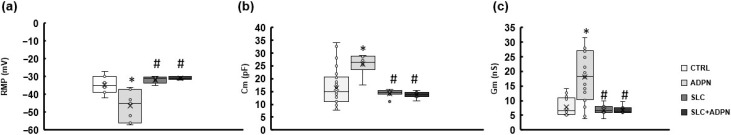
Involvement of SK2 in the action of ADPN in SMCs membrane properties. (**a**) Effect of adiponectin, ADPN (20 nM), of the sphingosine kinase 2 inhibitor SLC5111312 (SLC, 1 μM), and of SLC + ADPN on RMP values (in mV) of SMCs, compared to CTRL conditions. (*p* = 0.000197, one-way ANOVA with Bonferroni’s post hoc test). (**b**) Effect of ADPN, SLC, and SLC + ADPN on Cm (in pF) compared to CTRL condition (*p* = 0.00289, one-way ANOVA with Bonferroni’s post hoc test). (**c**) Effect of ADPN, SLC, and SLC + ADPN on Gm (in nS), compared to CTRL condition (*p* = 8.9 × 10^−6^, one-way ANOVA with Bonferroni’s post hoc test). Note the lack of effect of ADPN on passive properties of SMCs when added in the presence of SLC5111312. Data are as mean ± SD and values are listed in Table 1, together with *n* values. * *p* < 0.05 vs. CTRL; # *p* < 0.05 vs. ADPN.

Since we found that SK2 was the only isoform phosphorylated following ADPN treatment, we tested the possible involvement of this enzyme in ADPN-induced alterations of SMC bioelectrical properties. To this end we used the specific SK2 inhibitor SLC5111312 (SLC, 1 μM) as a selective pharmacological tool [27]. Using the microelectrode technique, we made electrophysiological records in murine gastric fundus strips starting from untreated preparations (CTRL) as described previously [13]. Successively, the sample was incubated with SLC5111312 added in the bath chamber. After 20 min the records were repeated. The addition of the SK2 inhibitor (SLC, Figure 3a–c) did not significantly alter any SMCs passive property compared to untreated samples (CTRL, Figure 3a–c), suggesting the reliability of this drug in our preparation. Finally, we added ADPN (20 nM) in the concomitant presence of the inhibitor and the recordings were achieved again after at least 20 min. We observed that ADPN added in the presence of SLC5111312 (SLC + ADPN) was no longer able to hyperpolarize the membrane, nor to alter the capacitance Cm and Gm of SMCs, in contrast to what we noted when the adipokine was applied alone (ADPN, Figure 3a–c). This result not only confirms the effect of ADPN on SMC function, but suggests a role of SK2 in the modulation of these rapid membrane phenomena induced by ADPN. All of the parameter values and the number of investigated SMCs (*n*) are listed in Table 1.

**Figure 4:**
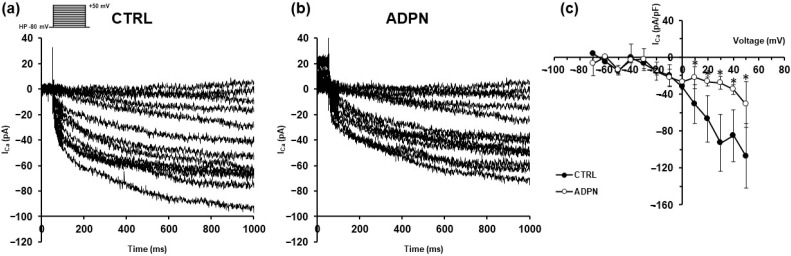
Adiponectin (ADPN) effects on Ca^2+^ currents recorded from murine gastric SMCs. (**a**) Representative inward slowly activating Ca^2+^ currents (in pA) evoked with the V-clamp step protocol (inset) in untreated sample preparation (CTRL). (**b**) Current records from the same preparation 20 min after the application of ADPN (20 nM). (**c**) Overall I–V plot (with current amplitude normalized for cell capacitance, pA/pF) showing data obtained in the presence of ADPN (*n* = 7) and in the absence of ADPN (CTRL, *n* = 7). Student’s *t* test, * *p* < 0.05 vs. CTRL.

We then examined the possible effects of these treatments on the transmembrane calcium current (I_Ca_), by applying the procedure described in Methods Section (Section 2) and the voltage pulse protocol shown in the inset of Figure 4. The amplitude of inward I_Ca_ recorded in untreated SMCs was reduced after ADPN addition confirming previous findings [12]. A representative example is shown in Figure 4, where the inward current recorded in CTRL (Figure 4a) shows a decreased amplitude in the presence of the adipokine (Figure 4b). This suggests that ADPN leads to a minor amount of Ca^2+^ ions for the Ca^2+^-dependent component of unitary SMC mechanical activity. The I–V relation related to all of the experiments performed (Figure 4c) confirmed the general behavior of the phenomenon.

**Figure 5:**
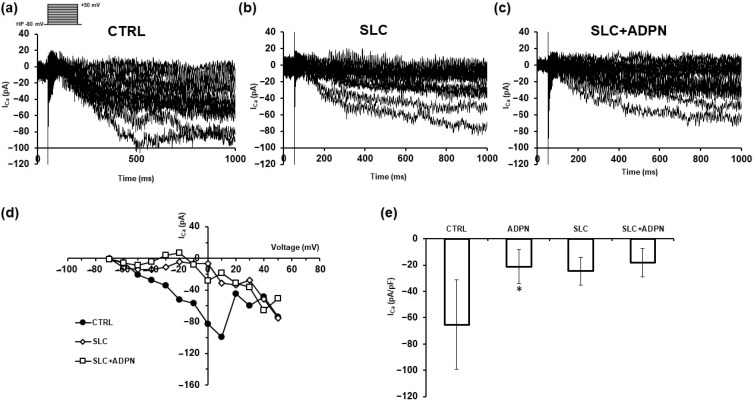
Outcome of SK2 inhibition in ADPN effects on Ca^2+^ ion currents recorded from murine gastric SMCs. (**a**) Representative current responses (in pA) evoked by the same pulse protocol reported in Figure 4a (and depicted in the inset) showing an inward slowly activating Ca^2+^ currents in untreated preparation (CTRL). (**b**) Current records from the same preparation, 20 min after the acute application of SLC (1 μM). (**c**) Currents recorded in the concomitant presence of SLC + ADPN. (**d**) I–V plot related to the currents shown in panels (**a**–**c**). (**e**) Bar charts related to the normalized Ca^2+^ peak current (in pA/pF) evoked by the +10 mV step pulse. Data are mean ± SD. CTRL (*n* = 5), ADPN (*n* = 5), SLC (*n* = 4) and SLC + ADPN (*n* = 4). One way ANOVA with Bonferroni post hoc test, * *p* < 0.05 vs. CTRL.

In a different set of experiments, we added SLC5111312 (SLC, 1 μM) to the bath medium. We observed that the current response in the presence of the inhibitor had a quite reduced size compared to its related CTRL (Figure 5a,b,d). This response was not further appreciably modified by the concomitant addition of ADPN (SLC + ADPN, Figure 5c,d). The general behavior of this phenomenon is reported in Figure 5e, where we show the mean normalized peak Ca^2+^ currents evoked by the +10 mV-voltage step pulse in the four different conditions: ADPN caused a significant reduction of the current amplitude compared to CTRL. The current size was reduced in the presence of the SLC alone and not significantly altered in the concomitant presence of SLC + ADPN. Our results may indicate that SK2 activity and the consequent amount of endogenous S1P produced can have a role in eliciting a consistent I_Ca_ following voltage dependent Ca^2+^ channels activation. Notably, the pharmacological blockade of this enzyme with the specific inhibitor prevents ADPN from exerting further appreciable effect on inward currents as those observed in Figure 4.

### 3.3. Cotreatment with ADPN and SK2 Inhibitor Modifies Myofilament Network Organization of the Contractile Apparatus Leading to a More Relaxed State

In the end, aiming to reveal possible changes of the contractile apparatus organization eventually induced by the different treatments, we performed a morphological analysis of CTRL-, ADPN-, SLC- and SLC + ADPN-treated gastric fundus muscle strips. The duration of the treatment was chosen on the basis of previous results, which demonstrated to cause an appreciable effect of ADPN both on mechanical and bioelectrical activity [11,18].

**Figure 6:**
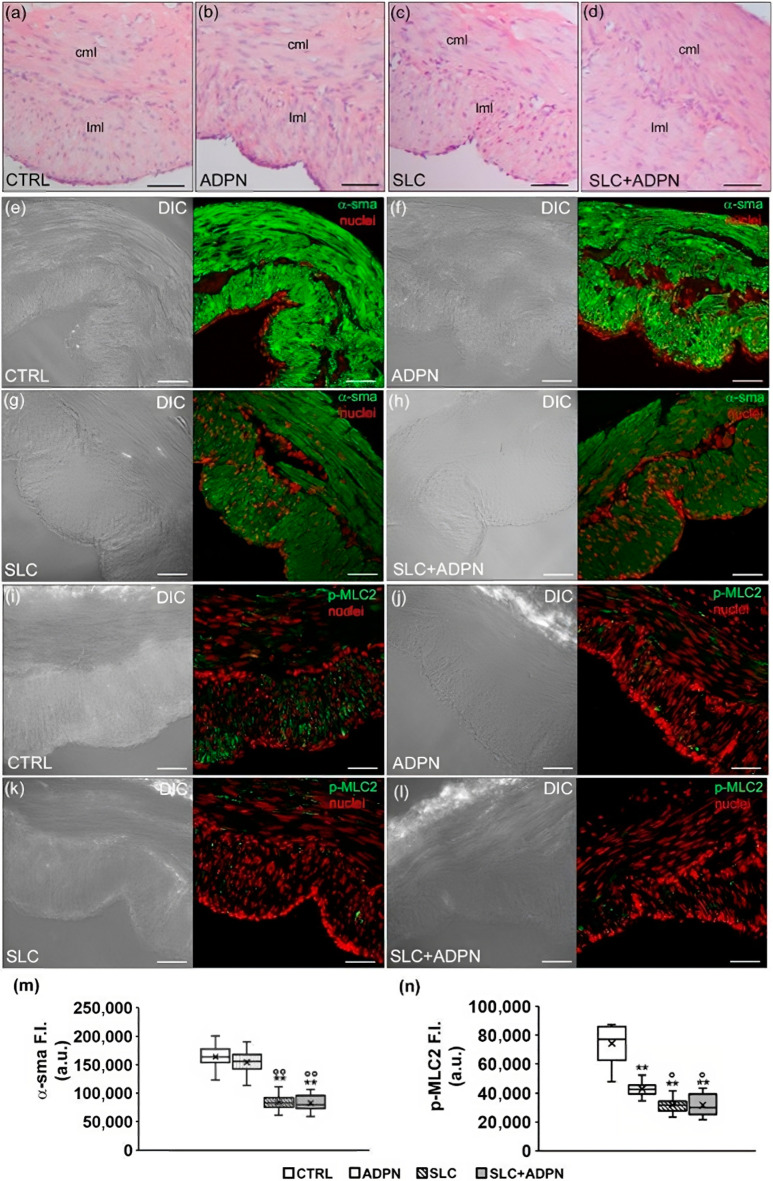
(**a**–**d**) Representative images (light microscopy) of sections of paraffin-embedded muscle strips from murine gastric fundus of control (CTRL, untreated), treated with adiponectin (ADPN), or with sphingosine kinase 2 inhibitor SLC5111312 in the absence (SLC) or presence of ADPN (SLC+ ADPN) stained with hematoxylin and eosin (H&E). cml = circular muscle layer, lml = longitudinal muscle layer. (**e**–**l**) Representative differential interference contrast (DIC, grey) and confocal immunofluorescence images showing α-smooth muscle actin (sma) staining ((**e**–**h**), green) and phospho-myosin light chain (p-MLC)2 ((**i**–**l**), green) Nuclei are put in evidence by PI in red. Scale bar 50 μm. (**m**,**n**) quantitative analyses of the fluorescence intensity (F.I.) in the arbitrary units (a.u.) of the indicated markers. ** *p* < 0.01 vs. CTRL; °° *p* < 0.01 vs. ADPN; ° *p* < 0.05 vs. ADPN (one-way ANOVA followed by Bonferroni’s post hoc test for multiple comparisons).

Light microscopic analysis of cross H&E-stained sections revealed no substantial differences in the structural features of all the analyzed muscle strips (Figure 6a–d). Indeed, we observed the two typical muscle layers in the gastric fundus wall with longitudinally and circularly arranged SMCs, in the external and internal layer, respectively. Nevertheless, confocal immunofluorescence analysis of the α-smooth muscle actin (sma), the predominant isoform composing the myofilaments of the contractile machinery within smooth muscle (Figure 6e–h,m), unravels a different staining of such protein among the samples. In particular, CTRL- and ADPN-treated strips showed an intense but not homogenous immunostaining. This may be consistent with a different myofiber cytoplasmic organization of α-sma in “thin and thick” aggregates (hence, lower and higher fluorescent signals, respectively), suggestive of a different arrangement of α-sma-containing thin myofilaments. In contrast, in SLC-treated samples, we observed a more homogeneous staining with the disappearance of the intensely fluorescent aggregates. This aspect is suggestive of a spatial reorganization of the contractile machinery as thin filament distancing occurring during SMC relaxation. The same observations were made for samples treated with SLC and ADPN, suggesting that the greater effect on this feature was due to SK2 inhibition. Furthermore, consistent with these observations, confocal immunofluorescence analysis of p-MLC2, correlated with myosin ATPase activity and SMC contraction, unraveled a reduced expression of such a protein in ADPN-treated muscle strips as compared to control; such a reduction appeared even more marked in SLC- and SLC + ADPN-treated samples (Figure 6i–l,n).

**Figure 7:**
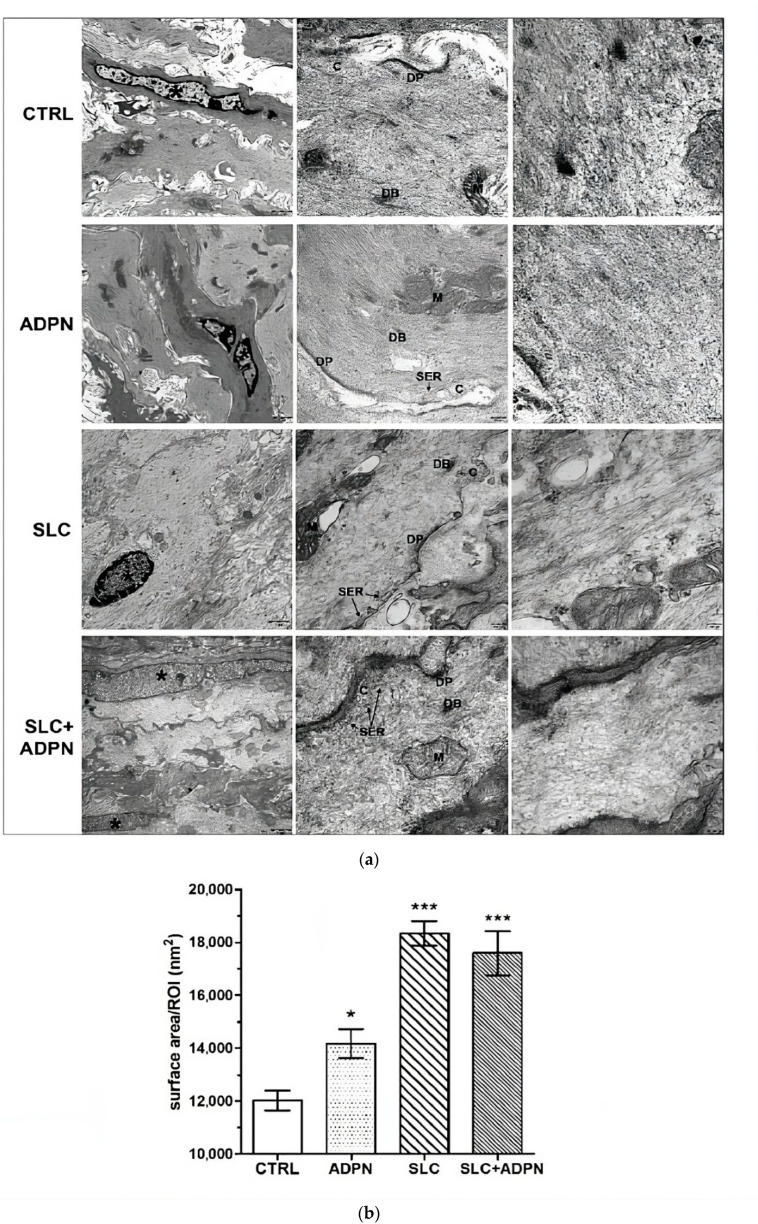
(**a**) Representative ultrastructural transmission electron microscopy (TEM) images of SMCs of the murine gastric fundus longitudinal muscle layer of the different indicated experimental groups. At low magnification (left panels), SMCs (nuclei labeled by asterisks) show no appreciable differences. At intermediate magnification (central panels), their cytoplasms show an extended framework of longitudinally oriented microfilaments with interposed dense bodies (DB) and converging to dense plaques (DP) at the inner aspect of the plasma membrane. In every group, the Ca^2+^-controlling system, made up of caveolae (C) and adjacent tubules of smooth endoplasmic reticulum (SER), and the mitochondria (M) show normal features. At high magnification (right panels), CTRL cell exhibits bundles of irregularly arranged actin contractile microfilaments; contractile filaments appear as a slightly more diffuse meshwork in ADPN-treated cell compared with the CTRL. The microfilaments appear to form a looser network in the SLC- and SLC + ADPN-treated cells. (**b**) The bar charts show the morphometrical analysis of relative area of the microfilament framework, evaluated on 20 regions of interest (ROI, 40,820 nm^2^ each) in high-magnification ultrastructural pictures of longitudinally sectioned SMC from the different experimental groups. * *p* < 0.05, *** *p* < 0.001 vs. CTRL (one-way ANOVA and Newman–Keuls multiple comparison post test).

Finally, to corroborate such suggestions, we performed an ultrastructural analysis, to better detect the contractile machinery of the SMC under the different treatments (Figure 7a,b). TEM images revealed that the SMCs of the different samples exhibited normal features of the typical structure of contractile machinery, namely extended framework of microfilaments with interposed dense bodies and converging to dense plaques at the inner part of the plasma membrane, the Ca^2+^ controlling system, made up of caveolae and adjacent tubules of smooth endoplasmic reticulum and the mitochondria. However the microfilament arrangement was different among the samples. In particular, ADPN-treated cells show a slightly looser network of contractile filaments, confirming the relaxant effects of ADPN on SMC cell activity. The microfilaments appeared arranged in more regular bundles and to form a less dense meshwork in the SLC- and SLC + ADPN-treated cells as compared to CTRL (Figure 7a,b). This aspect is likely due to increased dispersion of individual actin filaments from the microfilament bundles due to cell relaxation. Hence, we suggest that cotreatment with SK2 inhibitor and ADPN remodels myofilament network organization of the contractile apparatus, leading to a more relaxed state.

## 4. Discussion

The advancing research on adipokines and their mechanisms of action is of great interest to identify new therapeutic approaches or possible diagnostic/prognostic biological markers for diseases with altered energy balance. These pathologies are usually associated with adipose tissue depot alterations, hormone plasma level oscillations [32,33] and gastric modifications [34], a number of conditions that can ultimately affect the hunger–satiety cycle and feeding behavior.

For several years, many studies have tried to characterize the molecular mechanisms of action of one of these adipokines, ADPN, and the consequent signal transduction pathways activated [35,36], but to date, no unifying mechanism elucidates how ADPN can exert several different beneficial effects. It is well accepted that ADPN is mainly secreted by adipocytes of white adipose tissue, with blood levels inversely related to the fat mass, and targets many different cell types. Among them, hepatocytes, pancreatic β cells, endothelial cells and cardiomyocytes are worth noting [6]. The actions of ADPN [37] are supposed to be mediated by two well characterized and ubiquitously expressed related receptors, AdipoR1 and AdipoR2, and the coupled effector cascades. For instance, in relation with the ADPN role on energy balance, the classical target that seemed to be logically activated in the control of glucose homeostasis was shown to be the “key fuel sensing” kinase AMP-activated protein kinase (AMPK) [38]. About this, it was demonstrated that the binding of the hormone to its related receptors AdipoR1 and AdipoR2 actually activated AMPK. This, in turn, enhances glucose utilization, fatty acid oxidation arrests hepatic de novo lipogenesis and gluconeogenesis [39]. Similarly, the AMPK pathway was shown to be involved in another ADPN action, AdipoR1-mediated, that can be somehow related to energy homeostasis and feeding behavior, that is the modulation of gastric smooth muscle tone, considered a possible additional satiety signal [12]. Even if the most common signaling pathway is that mediated by AMPK in many preparations [35,40,41,42], different paths can be recruited by ADPN in other types of smooth muscle [43,44]. In this regard, our investigations are focused on deepening ADPN’s role in this context by studying other possible signaling pathways activated, to clarify the mechanism of action of this hormone. First of all, we here clearly demonstrated AdipoR1 and AdipoR2 expression in the murine gastric fundus muscular layer, in agreement with previous literature [13]. Then, since the pleiotropic actions of ADPN are reported to be triggered via receptor-mediated activation of ceramidase activity, we here considered for the first time in gastric preparations, the possibility that AdipoR are coupled with ceramidase activity, as demonstrated in other tissues [6,45,46].

In particular, it was documented that ADPN binding to its receptors induced the downstream activation of ceramidase, lowering ceramide levels [6,47]. As a consequence, the hydrolysis of ceramides produces sphingosine and free fatty acids. Thanks to SK activity, sphingosine can be phosphorylated to sphingosine 1-phosphate (S1P) that acts as a signaling molecule itself. S1P can act intracellularly or can be secreted out of the cell, exerting autocrine, paracrine or endocrine action. The compartmentalization of S1P is a key feature of this signaling molecule (in rodents and perhaps all vertebrates): intracellular S1P levels are low either because S1P is extruded by transporters, such as SPNS2, and/or it is degraded by S1P lyase or reversibly dephosphorylated by S1P specific phosphatase. Interestingly, S1P concentration in the blood and lymph is higher than in interstitial fluid, and this asymmetric distribution of S1P works as a signaling mechanism [48,49]. On these premises, we have here extended our preliminary investigations right in this direction, aiming to clarify with a pharmacological approach the possible involvement of the SK/S1P pathway as a further signaling pathway that mediates ADPN effect on gastric smooth muscle [50]. The outcomes of this research would add to the AMPK/NOS/NO/GC pathway previously identified [12,13,51], further indicating the extremely complex and multifaceted molecular mechanism of action of this peptide hormone.

According to previous findings [52], we revealed the presence of both SK1 and SK2 isoforms in our preparation. However, our novel results indicate that ADPN is able to increase the activation only of SK2. Similarly, in skeletal muscle myotubes, ADPN was recently shown to activate both isoforms of SK crucial for the adipokine-dependent modulation of electrophysiological properties and oxidative metabolism [46]. Here the ADPN-induced activation of SK2 but not SK1 supports the evidence of tissue-specific regulation of enzyme activity and ADPN action. The activation of SK2 induced by ADPN may lead to the increase of endogenous S1P production which, in turn, could possibly enhance the production of NO [53]. This is an interesting aspect that deserves to be verified in a dedicated study aimed to test NOS activation, in the absence or presence of the SK2 inhibitor, and hopefully to measure the effective NO production. At the moment, the pharmacological blockade of SK2 with SLC5111312 inhibitor, belonging to the class of inhibitors that are competitive with sphingosine [27], successfully prevented the very rapid effects of ADPN on RMP and bioelectric characteristics. With regard to Ca^2+^ currents, we confirmed that ADPN reduced its amplitude, further contributing to a prorelaxant action. This aspect, already tested by Garella and coll. [13], is also in line with previous literature showing the inhibition of calcium influx through voltage-gated calcium channels following the inactivation by NO/GC/cGMP/PKG axis [54]. Notably, the use of SLC5111312 caused in itself the reduction of the transmembrane calcium influx and a thin filament distancing, normally observed during SMC relaxation. The concomitant addition of ADPN in the presence of SLC5111312 did not significantly alter these phenomena, suggesting a prevalent effect of SK2 activity in controlling these electrical events and the asset of contractile filaments. In the present study, the reduction of Ca^2+^ currents observed in the presence of SLC5111312 alone may suggest that SK2 activity and the consequent amount of endogenous S1P produced, is necessary to observe a proper I_Ca_ appearance following voltage-dependent Ca^2+^ channels activation, SLC treatment alone being able to hinder, at least in part, I_Ca_ occurrence. Alternatively, we may think that SLC5111312 could somehow interact directly with the Ca^2+^ channels, as already reported on murine hippocampal neurons for another SK2 inhibitor, ABC294640 [55].

From the structural point of view, in agreement with a quiet, looser microfilament network, we also observed a reduced α-sma and p-MLC2 content in SLC-treated samples. Again, we may speculate that the addition of SLC5111312, causing the inhibition of SK2 activity, could lead to a reduced formation of endogenous S1P that normally has a procontractile action [52]. Since SK activity and the consequent S1P production is known to enhance α-sma production in smooth muscle cells [56], we can reasonably justify, at least in part, the observed decrease in α-sma in SLC-treated samples, regardless of the presence of ADPN.

Based on the overall morphological observations, we can suggest that the mild impact of ADPN observed on the contractile apparatus could be the result of the balance between the NO proreleasing action mainly triggered via AdipoR1 and that procontractile of S1P triggered via AdipoR2. The proreleasing action that is here supported by the observed inhibition of Ca^2+^ currents, together with the well-known NO-mediated signals that trigger the increase in myosin light chain phosphatase activity [57] contribute to a reduced SMC tone. On the other hand, the procontractile effect of S1P in SMC, that has already been reported although independently of ADPN’s action [58], causes an increase in MLC phosphorylation and, thus, in tension development [59]. In support of the idea of the two ADPN-induced pathways balancing each other, there is evidence that when the SLC-treated sample, where S1P formation is partly or likely hindered, was stimulated with ADPN, which can only activate the prorelaxing agent NO, a relaxed condition of the contractile machinery prevails.

To sum up, the preliminary results of this study demonstrate for the first time the involvement of the SK2/S1P pathway in the action of this adipokine on gastric SMCs in a murine model, suggesting a role of the bioactive lipid S1P in the ADPN-mediated control of gastric smooth muscle excitability and contractile machinery. In this regard, different studies have started to elucidate the composite signaling mechanisms by which S1P can modulate distinct biological functions such as cell growth, differentiation [60] survival, calcium homeostasis, and the regulation of many of the pathways that are also involved in smooth muscle [52,61]. Further investigations are needed to clarify in detail its possible crosstalk with other ADPN-activated pathways and to demonstrate the actual presence and the types of S1P specific receptors in our preparations, to corroborate the eventual autocrine/paracrine action of this bioactive sphingolipid.

Although with the limitations of an ex vivo study on a rodent animal model, these findings add a piece of knowledge to the action mechanisms of ADPN in gastric fundus SMCs. This clarification is necessary to consider this hormone and its receptors as new potential therapeutic targets for pathologies accompanied by altered energy balance and by adipose tissue mass alterations.

## Figures and Tables

**Table 1:** Effect of the different treatments on gastric fundus smooth muscle cells (SMCs) passive properties.

Parameter	CTRL	ADPN	SLC	SLC + ADPN
RMP (mV)	−34.6 ± 5.1 (*n* = 7)	−46.5 ± 8.6 * (*n* = 7)	−31.7 ± 1.9 # (*n* = 5)	−30.8 ± 0.8 # (*n* = 5)
Cm (pF)	16.4 ± 7.1 (*n* = 38)	25.6 ± 4.2 * (*n* = 6)	14.3 ± 1.6 # (*n* = 7)	13.6 ± 1.3 # (*n* = 7)
Gm (nS)	7.7 ± 3.8 (*n* = 13)	18.1 ± 8.7 * (*n* = 18)	6.7 ± 1.8 # (*n* = 7)	6.8 ± 1.4 # (*n* = 7)

Data are as mean ± SD. *n* is the number of analyzed SMCs. * *p* < 0.05 vs. CTRL; # *p* < 0.05 vs. ADPN (One-way ANOVA with Bonferroni’s correction).

## Data Availability

Data are available from the corresponding author on reasonable request.
